# *N*′-(5-Bromo-2-methoxy­benzyl­idene)-2-methoxy­benzohydrazide

**DOI:** 10.1107/S1600536809022971

**Published:** 2009-06-20

**Authors:** Xue-Song Lin, Ya-Li Sang

**Affiliations:** a Department of Chemistry, Chifeng University, Chifeng 024001, People’s Republic of China

## Abstract

The title hydrazone compound, C_16_H_15_BrN_2_O_3_, adopts an *E* configuration about the C=N double bond. The mol­ecule is twisted, the dihedral angle between the two substituted benzene rings being 22.0 (2)°. In the crystal structure, mol­ecules are linked through inter­molecular N—H⋯O hydrogen bonds, forming chains along the *c* axis.

## Related literature

For the biological properties of the hydrazone compounds, see: Khattab *et al.* (2005 ▶); Küçükgüzel *et al.* (2003 ▶); Çukurovalı *et al.* (2006 ▶). For the structures of hydrazone derivatives, see: Fun *et al.* (2008 ▶); Wei *et al.* (2009 ▶); Khaledi *et al.* (2008 ▶); Yang *et al.* (2008 ▶). For reference structural data, see: Allen *et al.* (1987 ▶).
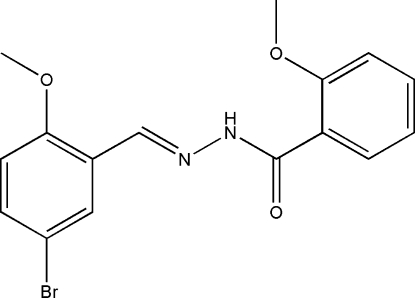


## Experimental

### 

#### Crystal data

C_16_H_15_BrN_2_O_3_
*M* *_r_* = 363.21
Monoclinic, 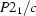

*a* = 13.3286 (3) Å
*b* = 11.4816 (3) Å
*c* = 10.1233 (2) Å
β = 99.128 (1)°
*V* = 1529.59 (6) Å^3^
*Z* = 4
Mo *K*α radiation
μ = 2.70 mm^−1^
*T* = 298 K
0.20 × 0.18 × 0.18 mm

#### Data collection

Bruker SMART CCD area-detector diffractometer
Absorption correction: multi-scan (*SADABS*; Sheldrick, 1996 ▶) *T*_min_ = 0.614, *T*_max_ = 0.642
9188 measured reflections
3323 independent reflections
2281 reflections with *I* > 2σ(*I*)
*R*_int_ = 0.025

#### Refinement

*R*[*F* ^2^ > 2σ(*F* ^2^)] = 0.038
*wR*(*F* ^2^) = 0.096
*S* = 1.01
3323 reflections
204 parameters
1 restraint
H atoms treated by a mixture of independent and constrained refinement
Δρ_max_ = 0.65 e Å^−3^
Δρ_min_ = −0.69 e Å^−3^

### 

Data collection: *SMART* (Bruker, 2002 ▶); cell refinement: *SAINT* (Bruker, 2002 ▶); data reduction: *SAINT*; program(s) used to solve structure: *SHELXS97* (Sheldrick, 2008 ▶); program(s) used to refine structure: *SHELXL97* (Sheldrick, 2008 ▶); molecular graphics: *SHELXTL* (Sheldrick, 2008 ▶); software used to prepare material for publication: *SHELXL97*.

## Supplementary Material

Crystal structure: contains datablocks global, I. DOI: 10.1107/S1600536809022971/at2816sup1.cif

Structure factors: contains datablocks I. DOI: 10.1107/S1600536809022971/at2816Isup2.hkl

Additional supplementary materials:  crystallographic information; 3D view; checkCIF report

## Figures and Tables

**Table 1:** Hydrogen-bond geometry (Å, °)

*D*—H⋯*A*	*D*—H	H⋯*A*	*D*⋯*A*	*D*—H⋯*A*
N2—H2⋯O2^i^	0.894 (10)	2.179 (19)	2.997 (3)	152 (3)

Symmetry code: (i) 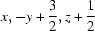.

**Fig. 1.**
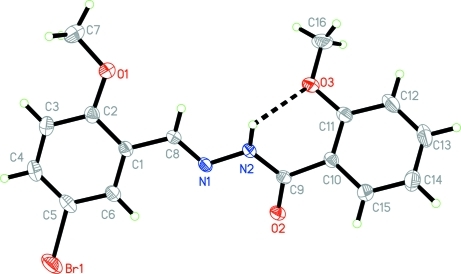
The molecular structure of (I), showing 30% probability displacement ellipsoids and the atom-numbering scheme.

**Fig. 2.**
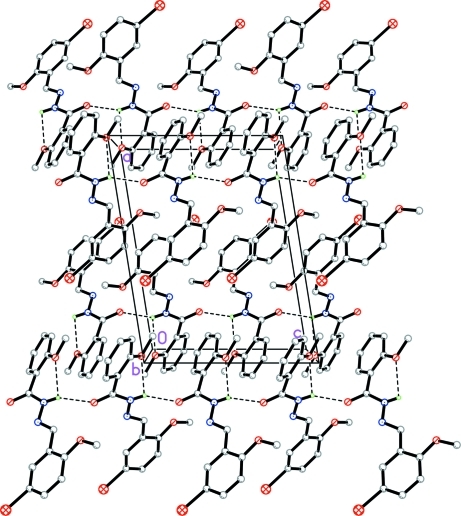
The crystal packing of (I). Hydrogen atoms not involved in hydrogen bonding have been omitted. Intermolecular hydrogen bonds are shown as dashed lines.

## supplementary crystallographic information

### Comment

Hydrazone and Schiff base compounds derived from the reaction of aldehydes with hydrazides have been widely investigated both for their crystal structures and biological properties (Khattab *et al.*, 2005; Küçükgüzel *et al.*, 2003; Çukurovalı *et al.*, 2006). In the last few years, a large number of hydrazone derivatives have been reported (Fun *et al.*, 2008; Wei *et al.*, 2009; Khaledi *et al.*, 2008; Yang *et al.*, 2008). However, the hydrazone compounds derived the 5-bromo-2-methoxybenzaldehyde have never been reported. In this paper, the crystal structure of the title new hydrazone compound, (I), derived from the reaction of 5-bromo-2-methoxybenzaldehyde and 2-methoxybenzohydrazide, is reported.

The molecular structure of (I) is shown as Fig. 1. The molecule adopts an *E* configuration about the C═N double bond. The molecule is twisted about the C8—N1—N2—C9 moiety, with the dihedral angle between the C1—C6 and C10—C15 benzene rings of 22.0 (2)°. All the bond lengths are within normal values (Allen *et al.*, 1987).

In the crystal structure of the compound, molecules are linked through intermolecular N–H···O hydrogen bonds (Table 1), forming chains along the *c* axis, as shown in Fig. 2.

### Experimental

5-Bromo-2-methoxybenzaldehyde (1.0 mmol, 215.0 mg) and 2-methoxybenzohydrazide (1.0 mmol, 166.2 mg) were mixed and refluxed in methanol (50 ml). The mixture was stirred for 1 h to give a clear colourless solution. Colourless crystals of (I) were formed by slow evaporation of the solution in air for a few days.

### Refinement

H2 attached to N2 was located in a difference map and refined with N–H distance restraint of 0.90 (1) Å. The other H atoms were positioned geometrically [*d*(C–H) = 0.93–0.96 Å], and refined using a riding model, with *U*_iso_(H) = 1.2*U*_eq_(C) and 1.5*U*_eq_(C_methyl_).

### Crystal data

**Table:**

C_16_H_15_BrN_2_O_3_	*F*(000) = 736
*M**_r_* = 363.21	*D*_x_ = 1.577 Mg m^−^^3^
Monoclinic, *P*2_1_/*c*	Mo *K*α radiation, λ = 0.71073 Å
Hall symbol: -P 2ybc	Cell parameters from 2540 reflections
*a* = 13.3286 (3) Å	θ = 2.4–27.4°
*b* = 11.4816 (3) Å	µ = 2.70 mm^−^^1^
*c* = 10.1233 (2) Å	*T* = 298 K
β = 99.128 (1)°	Block, colourless
*V* = 1529.59 (6) Å^3^	0.20 × 0.18 × 0.18 mm
*Z* = 4	

### Data collection

**Table:**

Bruker SMART CCD area-detector diffractometer	3323 independent reflections
Radiation source: fine-focus sealed tube	2281 reflections with *I* > 2σ(*I*)
graphite	*R*_int_ = 0.025
ω scans	θ_max_ = 27.0°, θ_min_ = 1.6°
Absorption correction: multi-scan (*SADABS*; Sheldrick, 1996)	*h* = −16→15
*T*_min_ = 0.614, *T*_max_ = 0.642	*k* = −14→12
9188 measured reflections	*l* = −12→12

### Refinement

**Table:**

Refinement on *F*^2^	Primary atom site location: structure-invariant direct methods
Least-squares matrix: full	Secondary atom site location: difference Fourier map
*R*[*F*^2^ > 2σ(*F*^2^)] = 0.038	Hydrogen site location: inferred from neighbouring sites
*wR*(*F*^2^) = 0.096	H atoms treated by a mixture of independent and constrained refinement
*S* = 1.01	*w* = 1/[σ^2^(*F*_o_^2^) + (0.0398*P*)^2^ + 1.0409*P*] where *P* = (*F*_o_^2^ + 2*F*_c_^2^)/3
3323 reflections	(Δ/σ)_max_ < 0.001
204 parameters	Δρ_max_ = 0.65 e Å^−^^3^
1 restraint	Δρ_min_ = −0.68 e Å^−^^3^

### Special details

**Table:**

Geometry. All e.s.d.'s (except the e.s.d. in the dihedral angle between two l.s. planes) are estimated using the full covariance matrix. The cell e.s.d.'s are taken into account individually in the estimation of e.s.d.'s in distances, angles and torsion angles; correlations between e.s.d.'s in cell parameters are only used when they are defined by crystal symmetry. An approximate (isotropic) treatment of cell e.s.d.'s is used for estimating e.s.d.'s involving l.s. planes.
Refinement. Refinement of *F*^2^ against ALL reflections. The weighted *R*-factor *wR* and goodness of fit *S* are based on *F*^2^, conventional *R*-factors *R* are based on *F*, with *F* set to zero for negative *F*^2^. The threshold expression of *F*^2^ > σ(*F*^2^) is used only for calculating *R*-factors(gt) *etc*. and is not relevant to the choice of reflections for refinement. *R*-factors based on *F*^2^ are statistically about twice as large as those based on *F*, and *R*- factors based on ALL data will be even larger.

### Fractional atomic coordinates and isotropic or equivalent isotropic displacement parameters (Å^2^)

**Table:**

	*x*	*y*	*z*	*U*_iso_*/*U*_eq_	
Br1	0.36301 (2)	1.06194 (4)	0.08660 (4)	0.07080 (16)	
O1	0.67425 (14)	1.12056 (19)	0.5818 (2)	0.0511 (5)	
O2	0.80411 (13)	0.73514 (17)	0.15200 (18)	0.0419 (5)	
O3	1.01119 (14)	0.80174 (18)	0.4696 (2)	0.0497 (5)	
N1	0.72756 (15)	0.88017 (18)	0.3201 (2)	0.0327 (5)	
N2	0.80859 (15)	0.80683 (19)	0.3608 (2)	0.0322 (5)	
C1	0.61419 (18)	1.0218 (2)	0.3825 (3)	0.0335 (6)	
C2	0.60017 (19)	1.1107 (2)	0.4729 (3)	0.0377 (6)	
C3	0.5157 (2)	1.1822 (2)	0.4485 (3)	0.0447 (7)	
H3	0.5068	1.2409	0.5089	0.054*	
C4	0.4447 (2)	1.1667 (3)	0.3350 (3)	0.0484 (7)	
H4	0.3873	1.2138	0.3194	0.058*	
C5	0.4594 (2)	1.0810 (3)	0.2451 (3)	0.0438 (7)	
C6	0.54289 (19)	1.0089 (2)	0.2674 (3)	0.0383 (6)	
H6	0.5516	0.9515	0.2054	0.046*	
C7	0.6646 (3)	1.2090 (3)	0.6772 (4)	0.0734 (11)	
H7A	0.6630	1.2839	0.6347	0.110*	
H7B	0.7214	1.2054	0.7483	0.110*	
H7C	0.6027	1.1974	0.7129	0.110*	
C8	0.70088 (18)	0.9428 (2)	0.4122 (3)	0.0340 (6)	
H8	0.7366	0.9383	0.4988	0.041*	
C9	0.84257 (17)	0.7374 (2)	0.2700 (2)	0.0300 (5)	
C10	0.93090 (18)	0.6607 (2)	0.3210 (2)	0.0331 (6)	
C11	1.01527 (19)	0.6938 (3)	0.4138 (3)	0.0376 (6)	
C12	1.0973 (2)	0.6182 (3)	0.4424 (3)	0.0517 (8)	
H12	1.1548	0.6414	0.5010	0.062*	
C13	1.0941 (3)	0.5100 (3)	0.3852 (3)	0.0588 (9)	
H13	1.1493	0.4600	0.4056	0.071*	
C14	1.0107 (3)	0.4748 (3)	0.2987 (3)	0.0576 (9)	
H14	1.0080	0.4002	0.2626	0.069*	
C15	0.9304 (2)	0.5502 (2)	0.2649 (3)	0.0447 (7)	
H15	0.8747	0.5267	0.2034	0.054*	
C16	1.0993 (2)	0.8449 (4)	0.5536 (4)	0.0690 (10)	
H16A	1.1117	0.7995	0.6342	0.103*	
H16B	1.0887	0.9248	0.5757	0.103*	
H16C	1.1568	0.8395	0.5076	0.103*	
H2	0.830 (3)	0.802 (3)	0.4490 (12)	0.080*	

### Atomic displacement parameters (Å^2^)

**Table:**

	*U*^11^	*U*^22^	*U*^33^	*U*^12^	*U*^13^	*U*^23^
Br1	0.0439 (2)	0.0908 (3)	0.0690 (3)	0.00890 (18)	−0.01763 (15)	0.0059 (2)
O1	0.0405 (11)	0.0619 (14)	0.0490 (12)	0.0068 (10)	0.0010 (9)	−0.0198 (10)
O2	0.0364 (10)	0.0531 (12)	0.0333 (10)	0.0071 (9)	−0.0033 (8)	−0.0053 (9)
O3	0.0324 (10)	0.0606 (14)	0.0520 (12)	0.0012 (9)	−0.0057 (9)	−0.0146 (10)
N1	0.0240 (10)	0.0347 (12)	0.0372 (12)	0.0022 (9)	−0.0016 (8)	0.0008 (10)
N2	0.0280 (10)	0.0361 (12)	0.0309 (11)	0.0053 (9)	−0.0006 (9)	−0.0003 (10)
C1	0.0268 (12)	0.0352 (14)	0.0387 (14)	0.0005 (10)	0.0050 (10)	0.0012 (11)
C2	0.0302 (13)	0.0409 (15)	0.0433 (15)	−0.0017 (12)	0.0103 (11)	0.0002 (12)
C3	0.0411 (15)	0.0387 (17)	0.0570 (19)	0.0061 (13)	0.0161 (14)	−0.0006 (14)
C4	0.0343 (14)	0.0463 (18)	0.066 (2)	0.0095 (13)	0.0111 (14)	0.0104 (15)
C5	0.0279 (13)	0.0518 (18)	0.0496 (17)	−0.0003 (12)	−0.0007 (12)	0.0129 (14)
C6	0.0322 (13)	0.0392 (15)	0.0424 (15)	0.0007 (12)	0.0022 (11)	0.0018 (13)
C7	0.066 (2)	0.086 (3)	0.067 (2)	0.002 (2)	0.0068 (18)	−0.036 (2)
C8	0.0280 (12)	0.0366 (15)	0.0354 (14)	0.0003 (11)	−0.0003 (10)	−0.0040 (12)
C9	0.0254 (11)	0.0327 (14)	0.0316 (13)	−0.0044 (10)	0.0030 (10)	0.0011 (11)
C10	0.0298 (12)	0.0402 (15)	0.0299 (13)	0.0040 (11)	0.0063 (10)	0.0027 (11)
C11	0.0309 (13)	0.0519 (18)	0.0302 (13)	0.0037 (12)	0.0055 (10)	0.0004 (12)
C12	0.0344 (15)	0.076 (2)	0.0432 (17)	0.0123 (15)	0.0004 (12)	0.0061 (16)
C13	0.0533 (19)	0.069 (2)	0.0544 (19)	0.0303 (18)	0.0104 (15)	0.0104 (18)
C14	0.067 (2)	0.0481 (19)	0.057 (2)	0.0191 (17)	0.0089 (17)	−0.0003 (16)
C15	0.0456 (16)	0.0454 (18)	0.0417 (16)	0.0073 (14)	0.0026 (12)	−0.0025 (13)
C16	0.0376 (17)	0.099 (3)	0.066 (2)	−0.0064 (18)	−0.0055 (15)	−0.033 (2)

### Geometric parameters (Å, °)

**Table:**

Br1—C5	1.902 (3)	C6—H6	0.9300
O1—C2	1.363 (3)	C7—H7A	0.9600
O1—C7	1.421 (4)	C7—H7B	0.9600
O2—C9	1.223 (3)	C7—H7C	0.9600
O3—C11	1.367 (3)	C8—H8	0.9300
O3—C16	1.425 (3)	C9—C10	1.495 (3)
N1—C8	1.273 (3)	C10—C15	1.390 (4)
N1—N2	1.380 (3)	C10—C11	1.398 (3)
N2—C9	1.348 (3)	C11—C12	1.389 (4)
N2—H2	0.894 (10)	C12—C13	1.369 (5)
C1—C6	1.390 (4)	C12—H12	0.9300
C1—C2	1.402 (4)	C13—C14	1.363 (5)
C1—C8	1.462 (3)	C13—H13	0.9300
C2—C3	1.383 (4)	C14—C15	1.377 (4)
C3—C4	1.380 (4)	C14—H14	0.9300
C3—H3	0.9300	C15—H15	0.9300
C4—C5	1.376 (4)	C16—H16A	0.9600
C4—H4	0.9300	C16—H16B	0.9600
C5—C6	1.376 (4)	C16—H16C	0.9600
			
C2—O1—C7	118.5 (2)	N1—C8—C1	120.2 (2)
C11—O3—C16	118.6 (2)	N1—C8—H8	119.9
C8—N1—N2	115.0 (2)	C1—C8—H8	119.9
C9—N2—N1	119.2 (2)	O2—C9—N2	123.0 (2)
C9—N2—H2	124 (2)	O2—C9—C10	120.7 (2)
N1—N2—H2	116 (2)	N2—C9—C10	116.3 (2)
C6—C1—C2	118.7 (2)	C15—C10—C11	118.3 (2)
C6—C1—C8	121.3 (2)	C15—C10—C9	116.3 (2)
C2—C1—C8	120.0 (2)	C11—C10—C9	125.3 (2)
O1—C2—C3	124.0 (3)	O3—C11—C12	124.2 (3)
O1—C2—C1	115.7 (2)	O3—C11—C10	116.3 (2)
C3—C2—C1	120.3 (3)	C12—C11—C10	119.5 (3)
C4—C3—C2	120.3 (3)	C13—C12—C11	120.5 (3)
C4—C3—H3	119.9	C13—C12—H12	119.7
C2—C3—H3	119.9	C11—C12—H12	119.7
C5—C4—C3	119.5 (3)	C14—C13—C12	120.6 (3)
C5—C4—H4	120.3	C14—C13—H13	119.7
C3—C4—H4	120.3	C12—C13—H13	119.7
C6—C5—C4	121.2 (3)	C13—C14—C15	119.7 (3)
C6—C5—Br1	119.3 (2)	C13—C14—H14	120.1
C4—C5—Br1	119.5 (2)	C15—C14—H14	120.1
C5—C6—C1	120.1 (3)	C14—C15—C10	121.3 (3)
C5—C6—H6	120.0	C14—C15—H15	119.4
C1—C6—H6	120.0	C10—C15—H15	119.4
O1—C7—H7A	109.5	O3—C16—H16A	109.5
O1—C7—H7B	109.5	O3—C16—H16B	109.5
H7A—C7—H7B	109.5	H16A—C16—H16B	109.5
O1—C7—H7C	109.5	O3—C16—H16C	109.5
H7A—C7—H7C	109.5	H16A—C16—H16C	109.5
H7B—C7—H7C	109.5	H16B—C16—H16C	109.5

### Hydrogen-bond geometry (Å, °)

**Table:**

*D*—H···*A*	*D*—H	H···*A*	*D*···*A*	*D*—H···*A*
N2—H2···O2^i^	0.89 (1)	2.18 (2)	2.997 (3)	152 (3)

Symmetry codes: (i) *x*, −*y*+3/2, *z*+1/2.
